# SPACA6-hosted miR-99b~125a~let-7e cluster shapes melanoma resistance by modulating mTOR-mediated immunosuppression

**DOI:** 10.3389/fimmu.2025.1719461

**Published:** 2026-01-02

**Authors:** Viviana Vallacchi, Gianpiero Lupoli, Eriomina Shahaj, Mariachiara Aloisi, Stefano Bergamini, Simona Frigerio, Loris De Cecco, Barbara Vergani, Katia Todoerti, Cristina Banfi, Biagio Eugenio Leone, Lorenza Di Guardo, Gianfrancesco Gallino, Mara Cossa, Barbara Valeri, Licia Rivoltini, Veronica Huber, Monica Rodolfo, Elisabetta Vergani

**Affiliations:** 1 Translational Immunology, Department of Experimental Oncology, Fondazione IRCCS Istituto Nazionale dei Tumori, Milan, Italy; 2 Integrated Biology of Rare Tumors, Department of Experimental Oncology, Fondazione IRCCS Istituto Nazionale dei Tumori, Milan, Italy; 3 Department of Medicine and Surgery, University of Milano-Bicocca, Monza, Italy; 4 Department of Diagnostic Innovation, Fondazione IRCCS Istituto Nazionale dei Tumori, Milan, Italy; 5 Unit of Functional Proteomics, Metabolomics and Network Analysis, Centro Cardiologico Monzino IRCCS, Milan, Italy; 6 Medical Oncology, Fondazione IRCCS Istituto Nazionale dei Tumori, Milan, Italy; 7 Melanoma Surgery, Fondazione IRCCS Istituto Nazionale dei Tumori, Milan, Italy

**Keywords:** melanoma, resistance, SPACA6, miR-99b~125a~let-7e cluster, PDE 3D models

## Abstract

Genomic, non-genomic, and immune alterations contribute to melanoma resistance to BRAF and MEK inhibitors. Here, we investigated the role of the SPACA6-hosted miR-99b~125a~let-7e cluster in modulating inflammatory processes and therapy resistance. We found that miR-99b, miR-125a, and let-7e were upregulated in progressing tumors from treated melanoma patients compared with untreated lesions, and in patients with short response duration compared with long-term responders. Similarly, miR-99b~125a~let-7e expression levels were high in melanoma cell lines with acquired resistance to BRAF/MEK inhibitors, showing upregulation of immunosuppressive cytokines. Combined inhibition of miR-99b, miR-125a and let-7e during drug treatment reduced proliferation of resistant cells and decreased the expression of pro-inflammatory cytokines such as CCL2, IL6, and IL8. Conversely, enforced overexpression of these miRNAs in drug sensitive cells promoted resistance and enhanced cytokine transcripts. In silico miR-99b, miR-125a and let-7e target gene analysis uncovered GNAI1, ADCY1 and NR6A1 genes in lipid metabolism pathways linked to BRAF/MEK inhibitor resistance, which converge on the activation of the mTOR signaling, and show down-regulation in resistant cells and tumors. RNA-seq and proteomic profiling of 3D cultures of patient-derived melanoma explants demonstrated that inhibition of the miR-99b~125a~let-7e cluster reprogrammed the tumor microenvironment, enhancing immune activation and suppressing mTOR signaling. Together, these findings identify the SPACA6-hosted miR-99b~125a~let-7e cluster as a regulator of BRAF/MEK inhibitor resistance through promotion of tumor survival and of an immunosuppressive microenvironment. Targeting this miRNA cluster may provide novel therapeutic opportunities to overcome drug resistance in metastatic melanoma.

## Introduction

Targeted therapy with BRAF and MEK kinase inhibitors (BRAF/MEKi) represents an essential treatment option for metastatic melanoma patients with BRAF mutated disease after progression on immunotherapy. However, drug resistance remains a clinical hurdle. Besides the spectrum of genetic alterations which are associated to BRAF/MEKi resistance, metabolic rewiring and profound epigenetic changes characterize melanoma plasticity and treatment resistance (1).

MicroRNAs (miRNAs) are main epigenetic modulators showing dysregulation in association with BRAF/MEKi resistance and with tumor-induced immune dysfunctions (2). Most miRNAs are clustered, located and co-expressed within host genes (3). miR99b~125a~let-7e cluster (NCBI Gene ID406910) resides in the host gene SPACA6, and the three miRNAs are simultaneously transcribed downstream (4). While SPACA6 has been assigned a role in gamete binding and fusion (5), miR-99b, miR-125a and let-7e were reported to regulate inflammatory immune responses and to be involved in autoimmune disease (6–8) and cancer biology (9–13). Besides a role as major regulators of hematopoiesis described in mice (14, 15), miR-99b, let-7e and miR-125a were shown to act as major regulatory elements of human blood monocytes differentiation toward an immunosuppressive anti-inflammatory cell polarization (16, 17), to display high expression in circulating CD14+ monocytes from metastatic melanoma patients (18), and to regulate monocyte trafficking and activation in the melanoma tumor microenvironment (TME) (19–23). In this context, Patient-Derived Explants (PDE) 3D cultures offer a valuable *ex vivo* model for investigating the effects of miRNA modulation in personalized cancer treatment, as they preserve the tumor’s original microenvironment and cellular heterogeneity (24, 25).

Here, we uncovered a role for SPACA6-hosted miR99b~125a~let-7e cluster in sustaining BRAF/MEKi resistance in melanoma tumors and cell lines. *In vitro* experiments in cell lines showed that these miRNAs regulate the release of factors promoting melanoma cell survival and drug resistance. GNAI1, ADCY1 and NR6A1 common target genes involved in the restriction of cAMP signal transduction and of the activation of mTOR pathway signaling were identified among the target genes in the lipid metabolism pathways dysregulated in resistant melanoma cells. Experiments in 3D cultures of melanoma PDE showed that simultaneous inhibition of miR-99b, miR-125a and let-7e affects the tumor immune microenvironment (TIME) while attenuating mTOR pathway activation.

## Materials and methods

### Melanoma tumor samples analysis

Formalin-fixed, paraffin-embedded (FFPE) tumor samples were collected from patients treated with BRAF/MEKi therapy at Fondazione IRCCS Istituto Nazionale dei Tumori (Milan, Italy). Patient sample studies were conducted with approval from the Independent Ethics Committee and the Institutional Review Board of Fondazione IRCCS Istituto Nazionale dei Tumori (INT39/11, INT40/11). All patients provided written informed consent. Samples were selected based on complete/durable or poor treatment response, as previously defined for RNA-seq study (26). miRNA expression profiling was carried using Agilent human microarrays miRBase v16. Data from the Agilent SureScan scanner and Feature Extraction software v10.7 were preprocessed with an optimized RMA algorithm (AgiMicroRna package) (27). miRNAs detected in at least one sample (gIsGeneDetected) were retained for downstream analysis. For qPCR studies, RNA was extracted with the NucleoSpin miRNA isolation kit (Macherey Nagel), quantified spectrophotometrically, and analyzed for expression levels of gene transcripts and miRNAs using reagents listed in **Supplementary Table 1**. GAPDH and RPL13A were used as endogenous controls for genes, U6 and SNORD48 snRNA for miRNA.

### Reagents and cell assays

Antibodies, probes, and oligos used throughout the study are listed in **Supplementary Table 1**. PLX4032 (Selleck) and Rapamycin (Biomol) was used at 3 μM and 1 μM, respectively. BRAF/MEKi-resistant melanoma cell lines, generated by repeated drug exposure (26), were compared with drug-sensitive parental cell lines, periodically checked for mycoplasma and authenticated by STR analysis (Gene Print 10 System, Promega). For transfections, cells were seeded at 8×10^5^ cells/well in 6-well plates, or 1.2×10^4^ cells/well in 96-well plates, and treated with 50 nM miRNA inhibitors or mimics, or with control oligos (Thermo Fisher Scientific) diluted in serum-free RPMI 1640 medium with Metafectene (Biontex); after 4h, medium with FCS was added and cells were analyzed 72h post-transfection. The generation of LM16 stable transfectants expressing CCL2 was previously reported (28). Cell proliferation was assessed at 72h after transfection in the presence/absence of PLX4032 or Rapamycin by using Cell Counting kit 8 (CCK8, Sigma), according to manufacturer’s instructions. The activation of caspase 3/7 upon 72h treatment with Rapamycin alone or combined with miRNA inhibitors or mimics was determined using luminescent Caspase Glo 3/7 assay (G8091, Promega). qRT-PCR was carried out in triplicate and run on QuantStudio 7 Flex instrument. Analysis was performed using SDS software, version 2.2.2. The results are presented as 2^−ΔCt^ ± SD for direct comparison. For flow cytometry analysis, APC- or PE-conjugated antibodies and corresponding isotype controls were used. Samples were acquired on a BD FACSCalibur flow cytometer (BD Biosciences) and analyzed with FlowJo v8.8.6. The gating strategy was generated by analyzing the data using Kaluza Analysis Software 2.1 (**Supplementary Figure 1**). Western blot analysis was carried as previously described (29).

### 3D cultures of melanoma PDE in RCCS bioreactor

PDE were prepared from fresh tumors as 3 mm biopsy punch cubes (Integra Miltex), cryopreserved in vitality freezing medium (RPMI1640 medium supplemented with 30% FCS and 10% DMSO), and cultured in duplicate in an RCCS bioreactor (Synthecon) in 2 mL RPMI1640 medium supplemented with 10% FCS added with either a pool of miR-99b, miR-125a, and let-7e inhibitors (50 nM each) or scrambled control (50 nM) oligos encapsulated in PLGA nanoparticles. After 72h, samples were recovered and split for RNA and protein analysis and fixed for histopathology. For immunohistochemistry, FFPE sections underwent antigen retrieval (0.5 mM EDTA pH 8 in a pressure cooker for 20 min) and staining with pAKT, pmTOR and mTOR antibodies using EnVision FLEX (Dako Agilent) with DAB chromogen (**Supplementary Table 1**). Slides were scanned on an Olympus BX63 microscope with DP80 camera and cellSens software (Shinjuku Monolith). For RNAseq, RNA was extracted by using Nucleospin miRNA kit (Macherey Nagel) and quantified using the Qubit RNA HS assay (Thermo Fisher Scientific), according to the manufacturer’s instructions. Libraries were prepared with the Illumina Stranded Total RNA protocol, and sequenced on a NovaSeq 6000 (Illumina) using an S2 flow cell (2×100 bp, stranded, 200 cycles). Differential expressed genes with FDR<0.05 and absolute log2 fold-change ≥ 0.5 were considered significant. Functional analyses of RNA-seq data were performed with Ingenuity Pathway Analysis (Qiagen). Cytokine profiling in PDE 3D culture supernatants was obtained by FirePlex Immunoassay by in service analysis (Abcam). For protein analysis of PDE tissues, protein lysates were obtained in RIPA buffer and quantified as previously described (29). Samples were diluted at 0.6 mg/ml and analyzed by Olink platform using Immuno-oncology panel (Olink Proteomics AB). Data were normalized using Olink’s proprietary normalization algorithm, including background subtraction and inter-plate calibration using internal controls.

### Statistical analysis

Statistical analyses were performed using GraphPad Prism software v.10. Continuous variables between two groups were compared using an unpaired two-tailed Student’s t-test or a Mann–Whitney U test, as appropriate. The Wilcoxon signed-rank test was used to compare patient-matched untreated and treated sample pairs. The correlation between linear variables was calculated using Spearman’s correlation coefficient. All *in vitro* experiments were repeated at least twice and a representative experiment is shown. Data are presented as the mean of three technical replicates ± standard deviation (SD).

## Results

### SPACA6 hosted miR-99b~125a~let-7e cluster is highly expressed in tumors of BRAF/MEKi-treated melanoma patients and associated with poor treatment response

**Figure 1:**
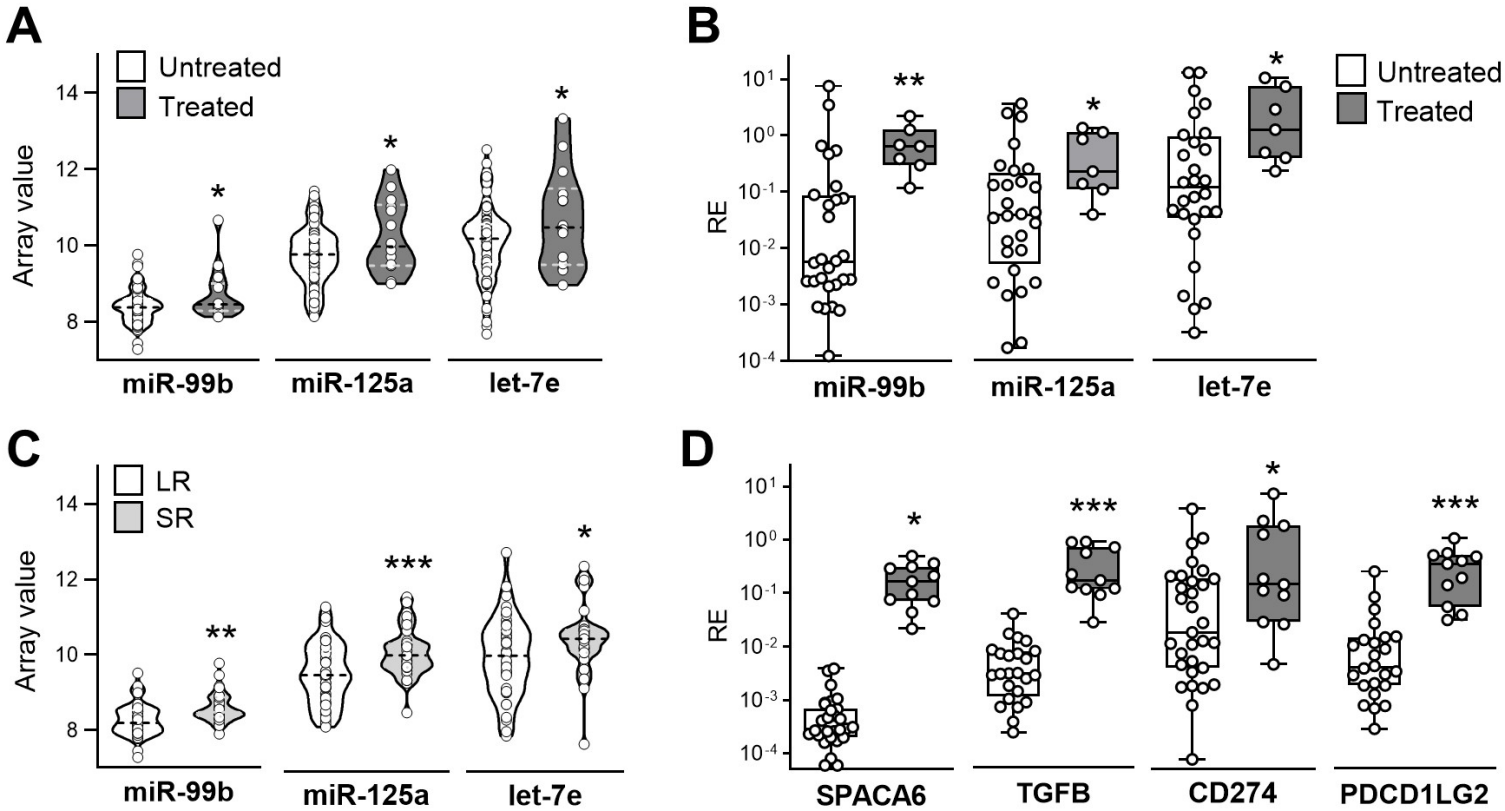
Melanoma from patients resistant to BRAF/MEKi treatment display enriched expression of the SPACA6 hosted miR-99b~125a~let-7e cluster and of TME-related immunosuppressive genes. **(A)** Expression levels of miR-99b, miR-125a, and let-7e in melanoma tumors excised from patients who progressed during BRAF/MEKi therapy (Treated, n=14) compared to tumor prior to therapy (Untreated, n=76) as resulting by miRNA profiling. **(B)** miRNA expression levels evaluated by qRT-PCR in an independent cohort of BRAF/MEKi-treated and untreated tumor samples (Treated n=11, Untreated n=31). **(C)** Expression levels of miR-99b, miR-125a, and let-7e in untreated tumors from long-term (LR, n=41) and short-term responders (SR, n=21) in the same sample set shown in A. **(D)**. qRT-PCR expression levels in the same sample set shown in **(B)** RE: Relative Expression. _*_: p<0.05, _**_: p<0.01, _***_: p<0.001 by Student’s unpaired t test (A, C) and by Mann-Whitney U t test (B, D).

Genetic and epigenetic alterations are associated with poor treatment response and development of drug resistance in metastatic melanoma. In a previous study we portrayed in detail the somatic alterations of resistant melanoma and the associated biological processes by their integration with transcriptional profiles in a retrospective series of tumors from resistant patients (26). miRNA profiling of this case set indicated a significant upregulation of miR-99b, miR-125a, and let-7e in progressing tumors from Treated patients compared to Untreated pre-therapy tumors, a pattern confirmed by qRT-PCR analysis (**Figures 1A, B**). Consistent with miR-99b, miR-125a, and let-7e localization as a cluster located in the host gene SPACA6, Treated tumors showed a higher SPACA6 gene expression compared to the Untreated samples (**Figure 1D**). These findings suggested that increased levels of miR-99b~125a~let-7e associate with the development of drug resistance upon BRAF/MEKi treatment. To analyze the potential association of a high tumor expression of the miR-99b~125a~let-7e cluster with clinical response to BRAF/MEKi treatment, we compared tumors excised pre-treatment from Long-term Responders (LR), i.e. patients which maintained treatment response for at least 2 years, and Short-term Responders (SR) from patients exhibiting a response duration of 6 months or less. The expression levels were higher in SR compared to LR samples (**Figure 1C**), indicating the association between a high expression of the miR-99b~125a~let-7e cluster with a reduced sensitivity to BRAF/MEKi of melanoma tumors.

As previously described, BRAF/MEKi resistance is characterized by an increased production of immunosuppressive cyto-chemokines such as TGFβ, IL6, IL8 and CCL2 (28, 30). This pattern includes the upregulation of immune checkpoints such as PD1, PDL1 and PDL2, contributing to the inhibition of effector T cell activity and to tumor progression (31). As the miR-99b~125a~let-7e cluster was reported to play a role in the regulation of PDL1 and PDL2 through the STAT3-SOCS1 signaling axis in monocytes activated *in vitro* by TLR (16), we hypothesized that they may be contributing to immunosuppression in the TME. We found that the upregulation of SPACA6 and of the hosted miRNAs in Treated *vs* Untreated tumor samples follows the same pattern as TGFB, CD274 (PDL1), and PDCD1LG2 (PDL2) genes (**Figure 1D**), thus suggesting a role for SPACA6-hosted miRNA cluster in the modulation of the TME, potentially influencing immune responses and therapeutic resistance in melanoma. These findings are consistent with our previous results showing the upregulation of miR-99b, miR-125a, and let-7e in blood monocytes exposed to melanoma released extracellular vesicles (18). These *in vitro* derived moMDSCs showed suppressive ability and a transcriptional gene signature detectable in advanced tumors (32, 33). Consistent to the higher expression of the miRNA cluster in Treated tumors, the moMDSCs gene signature displayed enrichment in Treated tumors compared to Untreated ones, and in Treated samples from SR patients poorly responsive to BRAF/MEKi when compared to responsive tumors from LR patients (**Supplementary Figures 2A, B**). Altogether these results showed that BRAF/MEKi resistance is sustained by an immunosuppressive TME associated to increased expression of miR-99b, miR-125a, and let-7e at the tumor site.

### Overexpression of miR-99b~125a~let-7e is associated with BRAF/MEKi resistance in melanoma cell lines

**Figure 2:**
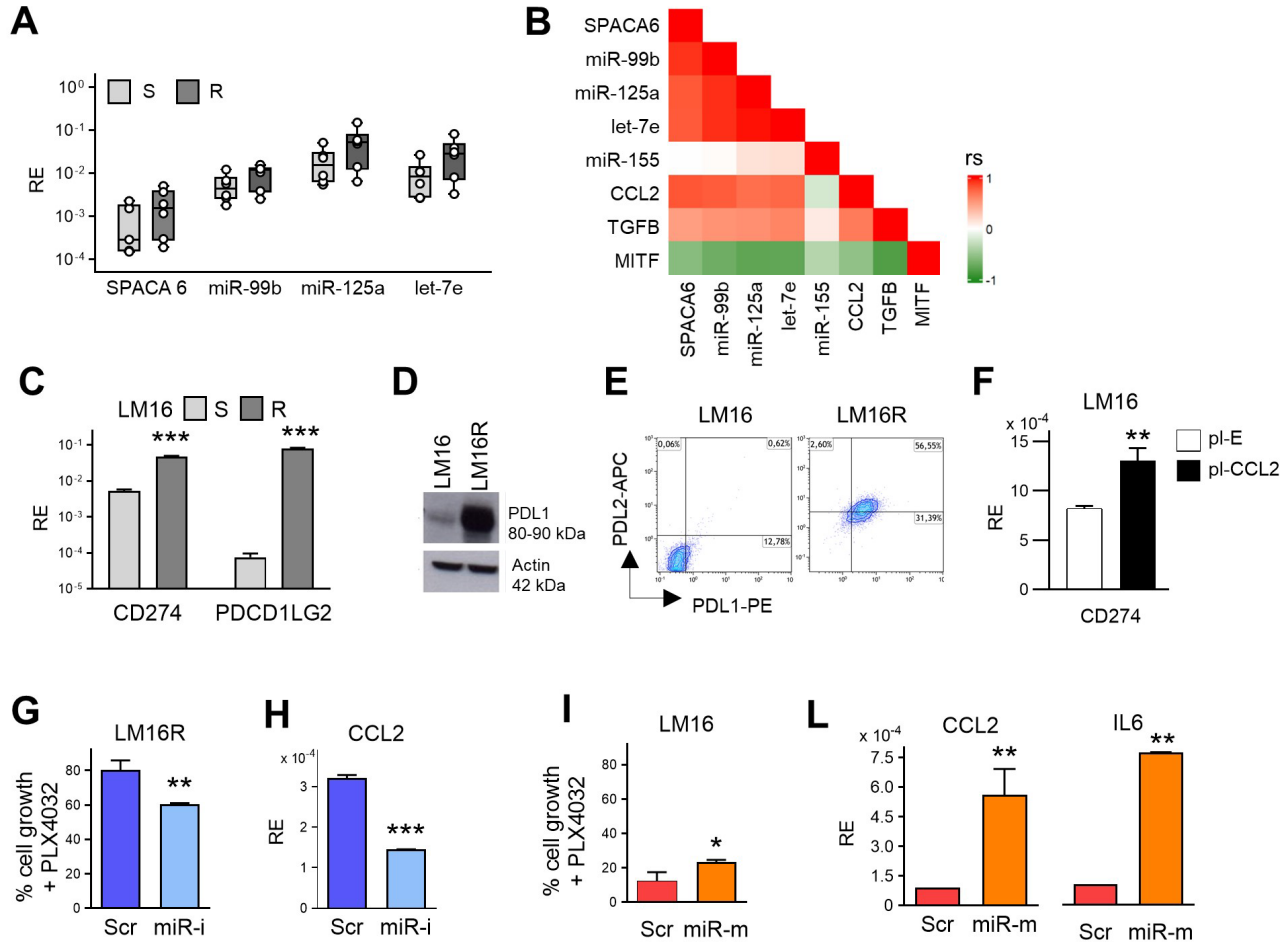
SPACA6-hosted miR-99b~125a~let-7e cluster affects melanoma cell proliferation in BRAFi resistance. **(A)** qRT-PCR analysis of SPACA6, miR-99b, miR-125a, and let-7e expression levels in BRAF/MEKi-resistant cell lines (R) compared to their sensitive counterparts (S). RE: Relative Expression. p<0.05 by Student’s unpaired t test in the overall comparison of S *vs* R groups. **(B)** Correlation plot showing positive correlations between the expression levels of SPACA6, miR-99b, miR-125a, let-7e, TGFB, and CCL2 in melanoma cell lines (Spearman p<0.05). MITF and miR-155 were included as negative controls. rs scale value is shown. **(C)** Expression levels of CD274 and PDCD1LG2 genes encoding for PDL1 and PDL2 in LM16 BRAF/MEKi-sensitive (S) and resistant (R) melanoma cell lines detected by qRT-PCR, **(D)** western blotting, and **(E)** flow cytometry. **(F)** Increased expression of CD274 gene in LM16 cell line transfected with CCL2 plasmid (pl-CCL2) in comparison to a control empty plasmid transfectant (pl-E). **(G)** Concomitant inhibition of miR-99b, miR-125a, and let-7e by synthetic inhibitors (miR-i) enhances the effect of treatment with PLX4032 in viability assay in LM16R resistant melanoma cells when compared with cell transfected with a scrambled control oligo (Scr). **(H)** Reduction of CCL2 expression levels following miRNA inhibition. **(I)** Forced overexpression of miR-99b, miR-125a, and let-7e by synthetic mimics (miR-m) reduced the antiproliferative effect of PLX4032 in LM16 cell line when assessed in comparison with scrambled negative control oligo (Scr). **(L)** Increased expression of CCL2 and IL6 transcripts in LM16 cells upon miR-99b, miR-125a, and let-7e overexpression by transfection synthetic mimics (miR-m). The modulation of miRNA expression levels upon the transfer of oligos is shown in **Supplementary Figure 4**, RE: Relative Expression. _*_: p<0.05, _**_: p<0.01, _***_: p<0.001 by Student’s unpaired t test (E-L).

To further support the observations obtained in tumors and to evaluate the role of miR-99b, miR-125a and let-7e in melanoma cells, we assessed their expression levels and that of the host gene SPACA6 in a panel of six BRAFV600E-mutated melanoma cell lines with acquired resistance to BRAFi and BRAF/MEKi previously characterized in our laboratory (26). Their mutational pattern showed enrichment in gene involved in oncogenic signaling pathways and in DNA repair, as also found in BRAFV600E melanoma cell lines resistant to BRAF inhibitor in the collection of the Cancer Cell Line Encyclopedia, and in tumors resistant to targeted therapy (26). In addition, these BRAF/MEKi-resistant cell lines displayed altered patterns of released immunomodulatory factors associated with pro-inflammatory and immunosuppressive functions, potentially contributing to tumor growth (28). qRT-PCR analysis showed that the three miRNAs were upregulated in resistant cell lines (R) compared to their original sensitive counterparts (S) (**Figure 2A**). A statistically significant difference was observed in the overall comparison of S *vs* R groups for SPACA6, miR-99b, miR-125a and let-7e. Considering all cell lines, the expression levels of the three miRNAs displayed a high positive correlation and were highly correlated to the expression of SPACA6; in addition, their expression levels were highly correlated to CCL2 and TGFB transcripts suggesting a common regulatory network (**Figure 2B**). Consistent with what observed in tumors, the expression of immune checkpoint PDL1 and PDL2 displayed upregulation in the resistant cell lines, as confirmed at gene and protein levels (**Figures 2C–E**; **Supplementary Figures 3A, B**). A common regulatory network driven by NF-κB transcriptional activity links CCL2 to PDL1 expression as shown following transfection of LM16 cells with a CCL2 expression plasmid (**Figure 2F**). These results indicated that also in melanoma cell lines resistant to BRAF/MEKi elevated levels of expression of miR-99b, miR-125a and let-7e were associated with increased expression of immunosuppressive factors sustaining a tumor-promoting microenvironment.

### Manipulation of miR-99b~125a~let-7e expression impacts on melanoma cell response to BRAFi treatment

To assess the potential role of the studied miRNAs in maintaining the viability and proliferation of resistant melanoma cells, we conducted loss/gain of function assays by transiently manipulating their expression using specific synthetic miRNA inhibitors or miRNA mimics. The concomitant inhibition of miR-99b, miR-125a, and let-7e significantly intensified the effects of treatment with the BRAFi PLX4032, as increased the reduction of viable cells in resistant cells (**Figure 2G**, **Supplementary Figures 3C, E**, **Supplementary Figure 4A**). In addition, the inhibition of miR-99b, miR-125a and let-7e levels reduced the expression of CCL2, IL6 and CXCL8 genes (**Figure 2H**, **Supplementary Figure 3D**). Conversely, enforced overexpression of miR-99b, miR-125a and let-7e by transfection of specific miRNA mimics in LM16 parental sensitive cells determined a detectable increase in cell proliferation upon BRAFi treatment when compared to the transfer of control oligos (**Figure 2I**, **Supplementary Figure 4B**). While this increase was limited (~11%), possibly due to the high sensitivity of LM16 cells to BRAFi, forced miRNA overexpression induced the upregulation of CCL2 and IL6 transcripts (**Figure 2L**). These results indicate that miR-99b~125a~let-7e contribute to the resistant phenotype of melanoma cells and link their expression to the production of immunosuppressive factors promoting melanoma cell growth, survival and drug resistance.

### miR99b∼125a∼let-7e cluster regulates genes in lipid metabolism pathways and mTOR pathway signaling

**Figure 3:**
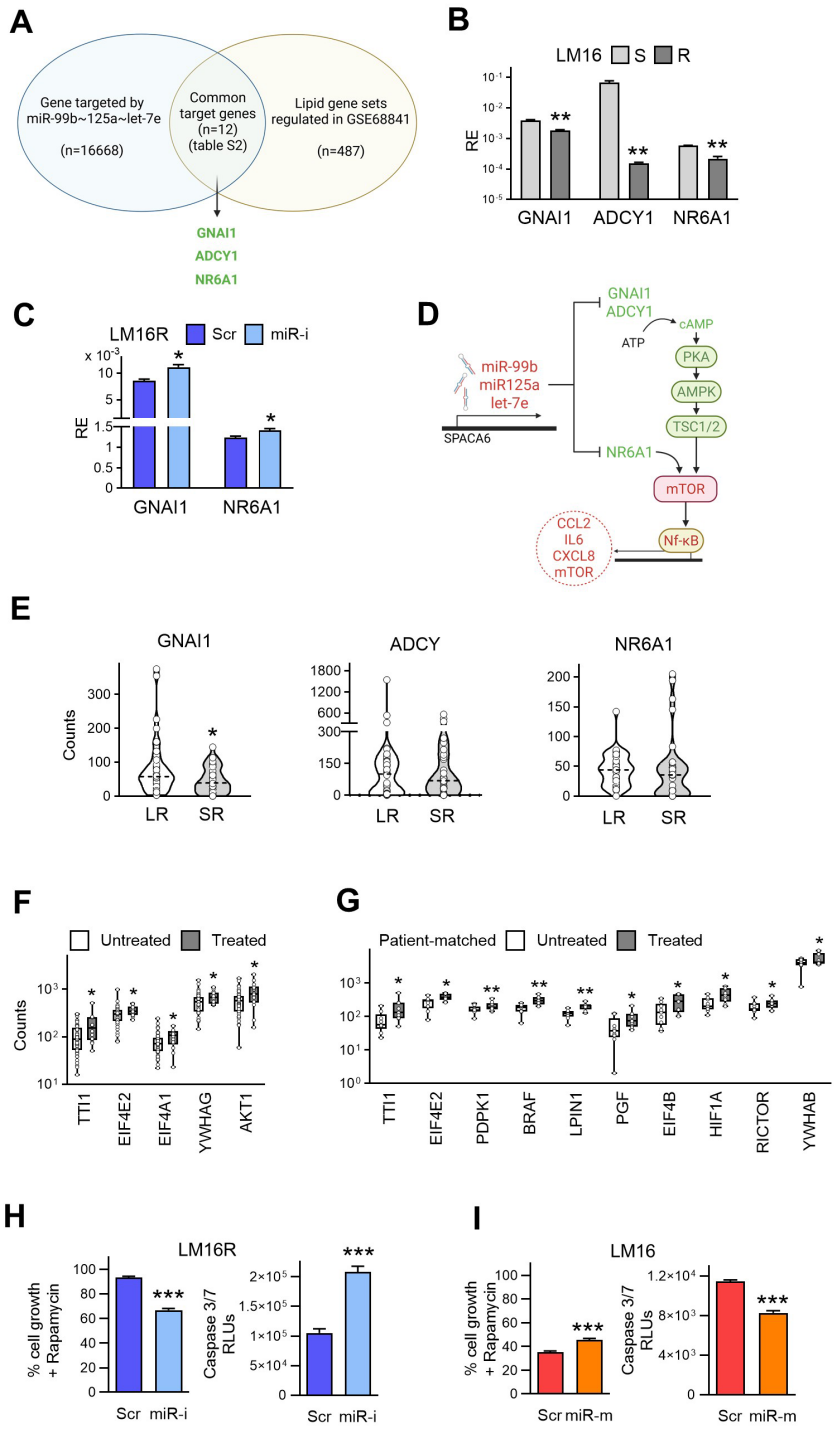
miR-99b~125a~let-7e cluster regulates lipid gene targets involved in the mTOR signaling pathway. **(A)** Diagram showing the identification of miR-99b~125a~let-7e downregulated common targets in lipid metabolism related-genes regulating mTOR. The resulting genes were obtained from the intersection between a list of 16,668 predicted target genes, obtained from miRWalk 3.0 and miRDB, with a list of 487 genes including the Hallmark Cholesterol Homeostasis gene set and GO terms for Cellular Response to Lipid and Fatty Acid Metabolism. **(B)** Expression levels of GNAI1, ADCY1 and NR6A1 in BRAF/MEKi-sensitive and -resistant LM16 melanoma cells. _**_: p<0.01 by Student’s unpaired t test. **(C)** Modulation of GNAI1 and NR6A1 gene expression upon concomitant inhibition of miR-99b, miR-125a, and let-7e by specific inhibitors (miR-i) compared to scrambled control (Scr). RE: Relative Expression. _*_: p<0.05 by Student’s unpaired t test. **(D)** Graphic representation of the miR-99b~125a~let-7e - mTOR axis via the regulation of common target genes GNAI1, ADCY1 and NR6A1. The miRNA cluster downregulates GNAI1, ADCY and NR6A1, which act at different levels of the mTOR signaling pathway, thereby resulting in the activation of the pathway and increase of tumor survival. Activation and reduction of mTOR-related proteins are indicated in red and green, respectively. **(E)** RNA-seq expression counts for GNAI1, ADCY1 and NR6A1 genes in melanoma untreated samples, stratified according to response to BRAF/MEKi. LR: long responders; SR: short responders. _*_: p<0.05 by Student’s unpaired t test. **(F)** Differential expression of genes associated to mTOR pathway considering Untreated and Treated tumor samples in the GSE196434 dataset. Genes were derived from a curated list of 126 mTOR pathway-associated from the mTOR pathway ontology and the Pathway Interaction Database for mTOR signaling pathway gene set. _*_: p<0.05 by Mann-Whitney U test. **(G)** Differential expression of genes associated to mTOR pathway considering a subset of 9 patient-matched Untreated-Treated sample pairs. _*_p<0.05, _**_ p<0.01 by Wilcoxon test. **(H)** Sensitivity of LM16R cells to Rapamycin (1 µM), alone or in combination with miRNA inhibitors (miR-i) as determined by CCK8 assay. Data are expressed as percent of cell growth compared to scrambled control (Scr) (left). Apoptosis induction after 72h treatment with Rapamycin (1 µM) alone or in combination with miRNA inhibitors as detected by caspase 3/7 activation assay (right). Data are expressed in Relative Light Units (RLUs). _***_: p < 0.001 by unpaired t test. **(I)** The same experimental setup was performed in LM16 cells using miRNA mimics (miR-m). _***_: p < 0.001 by Student’s unpaired t test.

In order to gain insights into the molecular pathways involved in drug resistance regulated by the miRNA cluster we analyzed their target genes. The miR99b∼125a∼let-7e cluster has been connected to broad metabolic pathways, including the regulation of pro-inflammatory cytokines and immune response mechanisms that are affected by lipid metabolism (34, 35). We focused the analysis on lipid metabolism pathways showing significant dysregulation in melanoma resistance to BRAF/MEKi in our previous studies. To evaluate the potential association of the studied miRNAs with the alterations observed in lipid metabolism pathways, we first obtained a list of their specific gene targets (n=16,668) by using two predicting algorithms, miRWalk 3.0 (36) and miRDB (37). In addition, a list of 487 genes was obtained from gene sets of GSEA Hallmark Cholesterol, GO Cellular Response to Lipid, and GO Fatty Acid Metabolism gene sets (28). The intersection of the two lists and the screening for gene targets common to the three miRNAs led to the identification of a list of 12 target genes (**Supplementary Table 2**), that included 5 genes showing downregulation in the LM16R *vs* LM16 dataset (GSE68841), and 3 of them, namely G Protein Subunit Alpha I1 (GNAI1), Adenylate Cyclase 1 (ADCY1), and Nuclear Receptor Subfamily 6 Group A Member 1 (NR6A1) displayed a significant negative fold change (**Figure 3A**). qRT-PCR analysis confirmed the significant downregulation of GNAI1, ADCY1, and NR6A1 genes in the resistant variants compared to their matched sensitive cell lines (**Figure 3B**, **Supplementary Figure 3F**). The simultaneous inhibition of the three miRNAs impacted the expression of GNAI1 and NR6A1 genes (**Figure 3C**), supporting a role for miR-99b∼125a∼let-7e cluster in regulating their expression.GNAI1 is a G protein that functions as a transducer downstream of G protein-coupled receptors, contributing to the regulation of ADCY1 signaling pathway (38). ADCY1 is a main regulator of the cAMP signaling pathway (39). Low levels of ADCY1 can determine low levels of cAMP, which lead to decreased levels of PKA and AMPK. This, in turn, inhibits TSC1/2 and activates the mTOR complex, which favors C/EBP-mediated transcription of genes promoting cell proliferation, migration and resistance to apoptosis (39). NR6A1 acts as a repressor of mTOR activity: low levels of NR6A1 contribute to mTOR activation, favoring the transcription of pro-tumor genes related to lipogenesis, angiogenesis, and NFkB signaling (40) (**Figure 3D**). To further support the role of the three miRNA-target genes in the regulation of mTOR signaling pathway, we evaluated their expression in a dataset of patient-derived samples from individuals treated with BRAF/MEKi with treatment response data (GSE196434). In tumors removed before treatment, GNAI1 was significantly downregulated in SR compared to LR patients, ADCY1 showed lower expression levels in SR, while no difference was observed for NR6A1 (**Figure 3E**). In tumors from Treated compared to Untreated patients several activators of mTOR signaling were deregulated. In fact, upregulation of TT1 (involved in the assembly and function of mTORC1), of EIF4E2, EIF4A1, YWHAG (coding for 14-3-3 proteins that bind several negative regulators of mTOR), and of AKT1 (an upstream regulator of mTOR) were shown (**Figure 3F**). The modulation of mTOR pathway was confirmed focusing the analysis on a subgroup of 9 patient-matched samples. Here, Treated samples display the upregulation of TT1, RICTOR, YWHAB, the downstream mTOR effectors EIF4E2 and EIF4B, the AKT activators PDPK1 and PGF, LPIN1 (a mTORC1 substrate), BRAF and HIF1A (**Figure 3G**).

These results link the support by miR-99b∼125a∼let-7e cluster to BRAF/MEKi melanoma resistance to the regulation of their direct target genes GNAI1, ADCY1 and NR6A1 involved in the activation of mTOR signaling pathway. To further investigate the role of the miRNA cluster in regulating activation of the mTOR signaling pathway, we treated the LM16R cell line with the mTOR inhibitor Rapamycin in combination with inhibition of miR-99b, miR-125a, and let-7e. This treatment resulted in a significant reduction in cell growth compared to the scrambled control. The effect was accompanied by increased apoptosis, as indicated by higher caspase-3/7 activity (**Figure 3H**). Conversely, in the sensitive LM16 cell line, the antiproliferative and pro-apoptotic effects of Rapamycin were attenuated by the transfection with miR-99b, miR-125a, and let-7e mimics (**Figure 3I**), supporting the association between the miRNA cluster and mTOR.

### miR-99b∼125a∼let-7e cluster manipulation in melanoma tumors regulates mTOR activation and condition TIME

**Figure 4:**
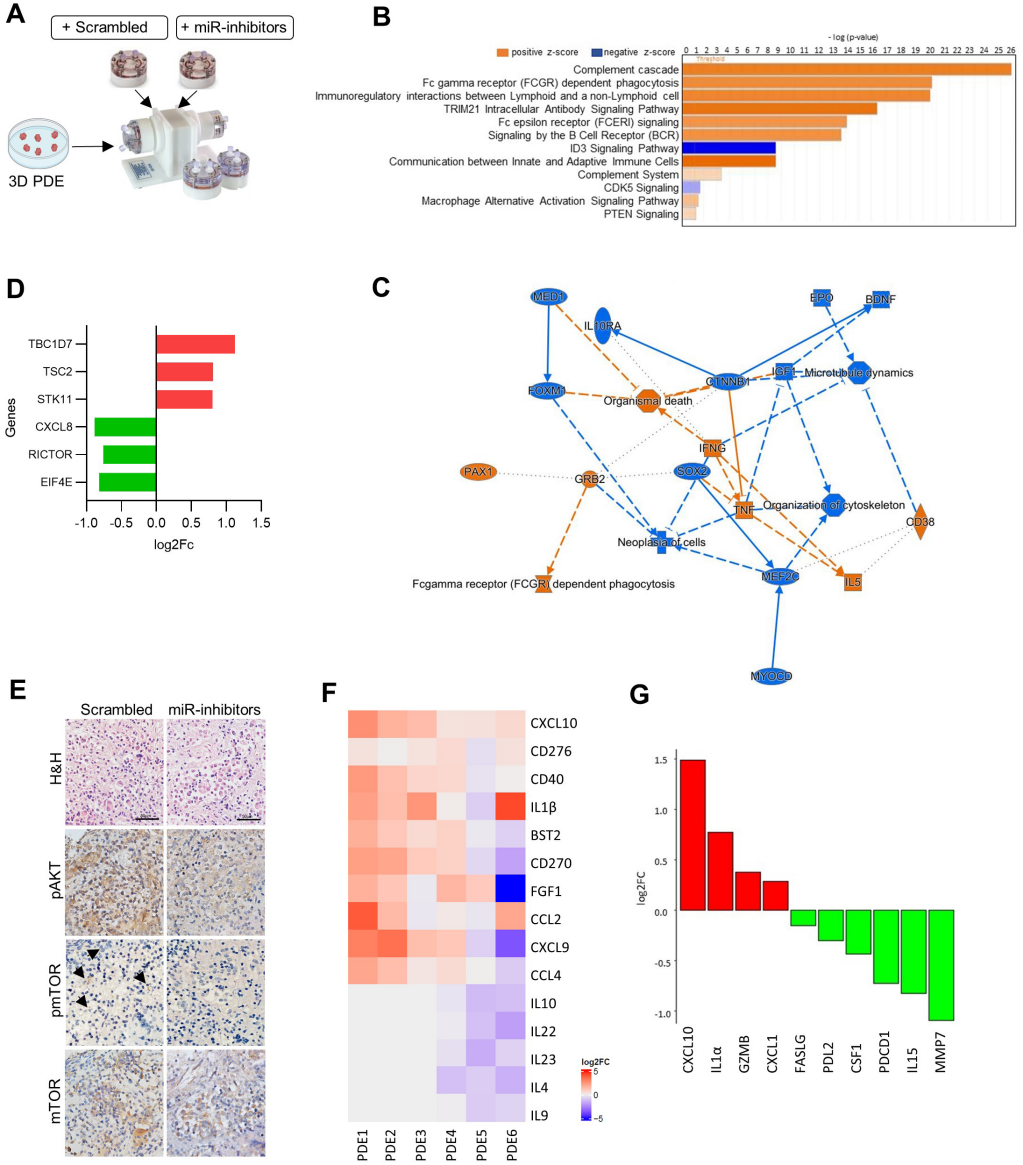
Inhibition of miR-99b~125a~let-7e reduces mTOR activation in melanoma PDE. **(A)** Schematic representation of 3D PDE culture experiments in RCCS Bioreactor. **(B)** Top canonical pathways identified by RNAseq using IPA software in significantly regulated genes in 3D PDE treated with miR-99b~125a~let-7e inhibitors compared to treatment with scrambled control. Upregulated pathways are in orange, downregulated pathways in blue. **(C)** Network representation of predicted functional categories and molecular interactions. Blue nodes and edges represent predicted inhibition, orange denotes activation; solid lines represent direct interactions while dotted lines indirect interactions. **(D)** Regulation of genes associated to mTOR pathway in 3D PDE treated with miR-99b~125a~let-7e inhibitors compared to scrambled control. Upregulated and downregulated genes are represented by red and green bars, respectively. **(E)** Immunostaining showing the downregulation of pAKT, pmTOR, and mTOR in representative treated PDE samples. Arrows indicate pmTOR positive cells. Scale bar: 10 μm. **(F)** Heatmap showing the soluble factors released by PDE in culture supernatants upon culture with miRNA inhibitors compared to scrambled control. Color intensity indicates the log2 fold change (log2FC) of treated versus control samples, with red representing increased secretion and blue decreased secretion relative to control condition. **(G)** Differential expression of proteins in PDE tissue lysates analyzed using the Olink proteomics platform. Protein abundance changes are represented as log2FC, with upregulated proteins shown in red and downregulated in green.

To evaluate the impact of miR-99b∼125a∼let-7e cluster inhibition in the regulation of the TME via mTOR signaling pathway, we conducted 3D experiments with melanoma PDE using a short-term dynamic culture system in a RCCS bioreactor, under conditions preserving the architecture and cellularity of the TME (25). PDE obtained from melanoma lesions excised from treatment-naïve patients were treated with inhibitors of the three miRNAs and analyzed after 72h culture (**Figure 4A**, **Supplementary Figure 4C**). Transcriptomic analysis of six different PDE cultures by RNA-seq identified 850 significantly regulated genes (p < 0.05) following inhibition of the miRNA cluster. Functional annotation with IPA revealed several top canonical pathways related to TME remodeling and immune activation. Upregulated pathways included the complement cascade, Fc gamma receptor–mediated phagocytosis, communication between innate and adaptive immune cells, and TRIM21 intracellular antibody signaling pathway, while ID3 signaling pathway, known to contribute to an immunosuppressive TME by promoting a Treg-like phenotype, showed downregulation. Additionally, regulation of the PI3K/AKT/mTOR axis was evidenced by the downregulation of CDK5 signaling, which is associated with its activation, together with the concomitant upregulation of PTEN signaling, its upstream negative regulator (**Figure 4B**). These pathways identified a gene network characterized by the upregulation of IFNG, TNF, IL5, and CD38, which stimulate the T cell compartment, and by the downregulation of IL10RA, IGF1 and CTNNB1, which are primarily associated with immunosuppression and cell proliferation (**Figure 4C**).

By interrogating mTOR-related genes, we found the downregulation of the mTOR pathway activators EIF4E, RICTOR, CXCL8 and the upregulation of the pathway inhibitors, such as STK11 encoding for LKB1 major upstream regulator, TSC2 and TBC1D7, a subunit of the TSC1-TSC2 complex (**Figure 4D**). Moreover, PDE treated with miRNA inhibitors downregulated mTOR signaling pathway, as displayed by reduced staining of pAKT, pmTOR and mTOR by immunohistochemistry analysis in comparison to control cultures (**Figure 4E**). Compared to control condition, the reduced miRNA levels were accompanied by increased released of several soluble factors related to immune activation, including the increase of CXCL10 (p=0.03), CD276, CD40, IL1β, BST2, indicating the activation of several immune cell populations (**Figure 4F**). Protein analysis of PDE samples confirmed that miRNA inhibition modulates TIME, as increased levels of CXCL10, IL1α, GZMB and CXCL1, and decreased levels of FASLG, PDL2, CSF1, PDCD1, IL15 and MMP7 were detected (**Figure 4G**). These results strongly support a central role for the miR-99b∼125a∼let-7e cluster/mTOR axis in modulating TIME.

## Discussion

In this study, we report for the first time that the SPACA6-hosted miR-99b~125a~let-7e cluster contributes to melanoma cell and tumor resistance to BRAF/MEKi through a mechanism involving the mTOR signaling pathway. SPACA6 gene is not recognized as a cancer driver, although its expression has been detected in multiple cancer types (10, 41). We observed elevated SPACA6 expression levels in melanoma tumors that progressed during BRAF/MEKi therapy, an increase associated to the upregulation of the hosted miR-99b∼125a∼let-7e cluster. Notably, this expression pattern correlated with poor clinical outcome after BRAF/MEKi therapy, and the different expression levels of the miRNAs between tumors from responders and refractory patients suggested their potential association with treatment response. Concomitant with the upregulation of SPACA6 levels, elevated expression of the miR-99b∼125a∼let-7e cluster was reported in liver cancer and esophageal squamous cell carcinomas, where it promoted tumor invasion and metastasis through regulation of ZEB1 activity (11, 12, 42). On the contrary, in pancreatic cancer and glioma, these miRNAs were shown to suppress tumor proliferation by targeting NR6A1 and GAS5 (10, 43). Beyond the association of SPACA6-hosted miRNA cluster with tumor progression, we detected a positive correlation between SPACA6, miR-99b, miR-125a and let-7e with key immunosuppressive factors such as TGFβ and CCL2, suggesting a potential role in sustaining an immunosuppressive TME. Moreover, our study indicates that the miR-99b~125a~let-7e cluster is associated with the accumulation of moMDSC in treated resistant tumors, an observation consistent with previous reports highlighting the role of MDSCs in establishing an immunosuppressive TME (44). The miR-99b~125a~let-7e cluster has been shown to mediate TLR-induced IL-6 production by targeting the MAPK inhibitor TRIB2, ultimately promoting cytokine receptor–dependent STAT3 activation (16, 17, 45). miR-99b has also been reported to suppress M2 macrophage polarization by repressing the mTOR/IRF4 axis, leading to tumor regression characterized by increased CD8^+^ T-cell infiltration and reduced MDSC and Treg populations in hepatocellular carcinoma (13). These findings point to a dual role of the miRNA cluster, not only promoting tumor cell survival but also shaping a TME conducive to immunosuppression, potentially further contributing to therapeutic resistance.

*In vitro* melanoma cell lines with acquired resistance to BRAF/MEKi showed upregulation of miR-99b~125a~let-7e cluster and SPACA6 compared to their sensitive counterparts, and the concomitant inhibition of miR-99b, miR-125a, and let-7e increased cell sensitivity to BRAFi, demonstrating their direct involvement in drug resistance. Moreover, their inhibition led to a reduction in key pro-inflammatory cyto-chemokines genes, such as CCL2, IL6, and CXCL8, regulated by NFkB-mediated transcription and contributing to melanoma cell survival and to immunosuppressive TME (28). This aligns with previous studies suggesting that inflammatory pathways regulated by NFkB are enriched among the predicted targets of the miR-99b~125a~let-7e cluster (16, 17, 45). Notably, the enforced expression of miR-99b, miR-125a and let-7e increased the production of these cytokines in sensitive cells, reinforcing the link between the miRNAs and the NFKB-mediated transcription of immunosuppressive released factors. These results demonstrate a functional relevance of the miRNA cluster in sustaining BRAF/MEKi resistance.

Lipid metabolism pathways revealed significant dysregulation in melanoma progression (46) and resistance to BRAF/MEKi (29, 47, 48). The studied miRNAs were found to impact on the expression of GNAI1, ADCY1, and NR6A1 common putative target genes in lipid metabolic pathways, involved in mTOR pathway activation. GNAI1 belongs to the Gαi family of G proteins and plays a role in signal transduction pathways principally as an inhibitor of adenylyl cyclase (49). Reduced GNAI1 expression has been reported in hepatocellular carcinoma, where it is associated with increased tumor cell migration and invasion (49), as well as in colorectal cancer metastasis, where its loss enhances angiogenesis through activation of JAK2/STAT3 signaling and upregulation of VEGFA (50). Sun et al. demonstrated that GNAI1 knockout abolished VEGF-induced Akt-mTORC1 and Erk activation in mouse embryonic fibroblasts (51). GNAI1 expression has also been reported to promote the growth and invasion of breast, nasopharyngeal, pancreatic, and glioma cancer cells through activation of the ERK/AKT/mTORC1 pathway (52–57). Moreover, high GNAI1 levels have been associated with an increased risk of relapse in HER2-positive patients treated with trastuzumab (58). Functionally, ADCY1 catalyzes ATP to cyclic adenosine 3’, 5’-monophosphate (cAMP). As a second messenger, cAMP regulates its downstream effectors to influence DNA damage responses, cell apoptosis and cell proliferation (39). In lung cancer, ADCY1 was significantly associated with platinum-based chemotherapy resistance by regulating Bcl-2-mediated apoptosis (59). High levels of ADCY1 was associated with poor overall survival in patients with metastatic melanoma, glioma, lung and renal tumors (60–63) but was found downregulated in prostate cancer, osteosarcoma, rectal adenocarcinoma metastasis and pancreatic adenocarcinoma (64–67). NR6A1 gene was associated with migration and invasion in prostate cancer (68). In addition, NR6A1 was shown to regulate lipid metabolism, insulin-induced proliferation and cell migration in HepG2 hepatocellular carcinoma cells, by acting through AKT/mTORC1 pathway (40).

The direct evidence that the studied miRNAs regulate the genes identified by in silico screening and their direct mechanistic involvement is not provided in this study, and remains to be demonstrated. Here, we speculate that the upregulation of miR-99b, miR-125a and let-7e represents an epigenetic mechanism sustaining BRAF/MEKi resistance through the downregulation of GNAI1, ADCY1, and NR6A1 genes, involved in the activation of mTOR signaling pathway and consequently to the NFkB high transcriptional state, positively regulating CCL2, IL6 and CXCL8 expression (**Figure 3D**). mTOR/NFkB axis has been previously identified as downstream target of miR-99b (13). Notably, the inhibition of the miRNAs in the resistant cell lines increased the expression of GNAI1 and NR6A1 and reduced melanoma cell growth. The activation of the mTOR pathway mediated by GNAI1, ADCY1, and NR6A1 was also evident in SR compared to LR patients upon BRAF/MEKi treatment, in line with our previous findings showing the enrichment of mTORC1/NFkB signaling in tumors from poorly responsive patients and in resistant tumors excised from treated patients (26). Recently, mTORC1 activation has been reported as a resistance mechanism in BRAF-mutant melanoma, and combining BRAF/MEKi with the mTORC1 inhibitor rapamycin was shown to effectively target resistant melanoma cells (69). Moreover, our *in vitro* findings suggest that this miRNA cluster plays a critical role in modulating mTOR signaling. In LM16R cells, inhibition of miR-99b, miR-125a, and let-7e enhanced the response to Rapamycin, resulting in reduced cell growth and increased apoptosis.

Functional studies using 3D melanoma PDE cultures in bioreactor, in which miR-99b, miR-125a and let-7e were simultaneously inhibited, further validated their impact in triggering the mTOR pathway promoting an immunosuppressive TME. Transcriptomic analysis revealed the upregulation of genes associated with canonical pathways linked to immune activation, together with the downregulation of genes driving pro-survival mTOR signaling. These molecular alterations were further supported by proteomic profiling of PDE and of the secreted factors, which highlighted a shift in the TIME from an immunosuppressive toward an immunostimulatory state. Consistently, miRNA inhibition reduced the levels of pAKT, pmTOR, and total mTOR by immunohistochemical analysis.

In conclusion, our study uncovers the critical role of the SPACA6-hosted miR-99b~125a~let-7e cluster in sustaining BRAF/MEKi resistance in melanoma. Through the activation of the mTOR signaling pathway and modulation of an immunosuppressive TME, this miRNA cluster promotes tumor survival and drug resistance. Targeting these miRNAs may represent a strategy to improve the efficacy of BRAF/MEKi therapy in melanoma patients by modulating the TME. In addition, our data further support the therapeutic potential of targeting molecular components involved in mTOR pathway regulation and emphasize the importance of developing next-generation agents capable of modulating this pathway with greater precision and reduced toxicity - such as nanoparticles designed to encapsulate both miRNAs and mTOR inhibitor.

## Data Availability

The data presented in the study are deposited in the GEO repository, accession number GSE312671.
