# Supplementary material for: SPACA6-hosted miR-99b~125a~let-7e cluster shapes melanoma resistance by modulating mTOR-mediated immunosuppression
Source: Front Immunol. 2026 Jan 2;16:1719461. doi: 10.3389/fimmu.2025.1719461 (PMC12808473; doi:10.3389/fimmu.2025.1719461)
Supplement: Supplementary file 1 [file DataSheet1.pdf]

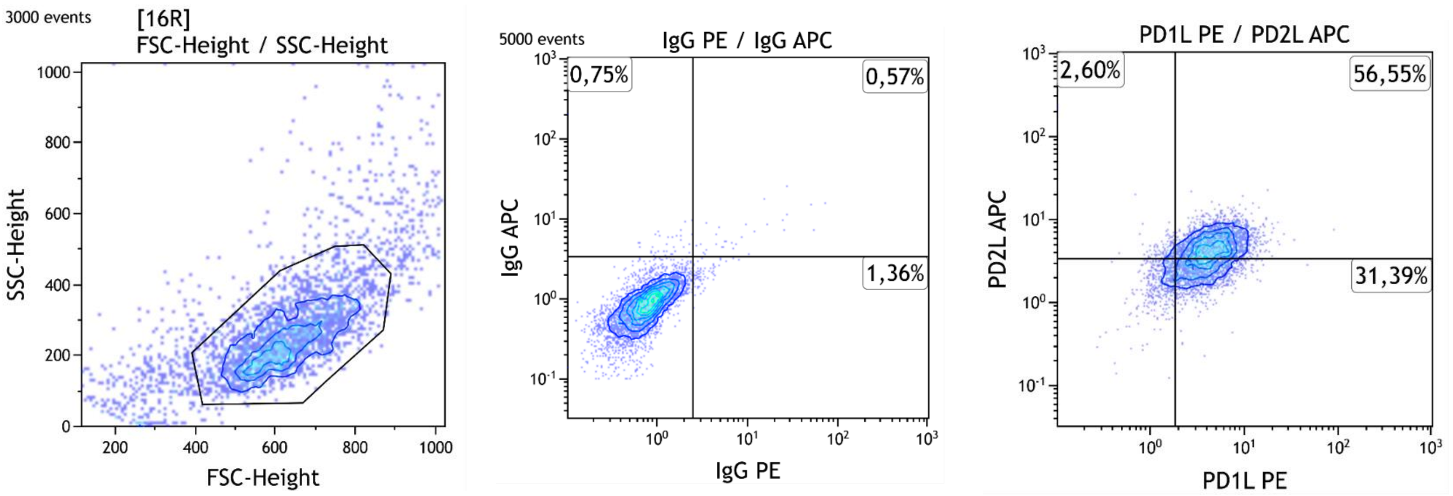

Supplementary Figure 1. Gating strategy applied to define PDL1 and PDL2 levels in BRAF/MEKi sensitive and resistant melanoma cell lines. Events were acquired by BD FACSCalibur flow cytometer (BD Biosciences) and analysed by Kaluza analysis software 2.1.

Supplementary Figure 1

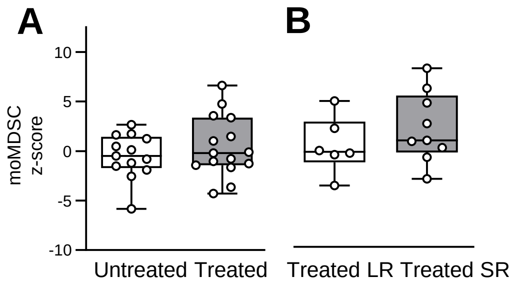

Supplementary Figure 2. Modulation of moMDSC gene signature in association to BRAF/MEKi Treatment and patient response. **(A)** Box plots showing z-scores of the moMDSCs gene signature in the GSE196434 dataset between Untreated (n=13) and Treated (n=15) tumors, and **(B)** in Treated tumors from long-term responders (LR, n=6) and short-term responders (SR, n=9). Z-scores were calculated as the sum of the standardized expression values of the genes in the gene set, divided by the square root of the number of genes by the gsva function in the v 2.0.7 GSVA package (doi:10.1186/1471-2105-14-71). Samples were stratified in high or low z-score group, according to median z-score value. High and low z-score groups were visualized by means of ggboxplot function from ggpubr package version v 0.6.0 (https://rpkgs.datanovia.com/ggpubr/2) in R environment (v 4.4.3). p=0.1 by Wilcoxon test and multiple mean comparisons, comparing means function and holm p-value adjustment.

Supplementary Figure 2

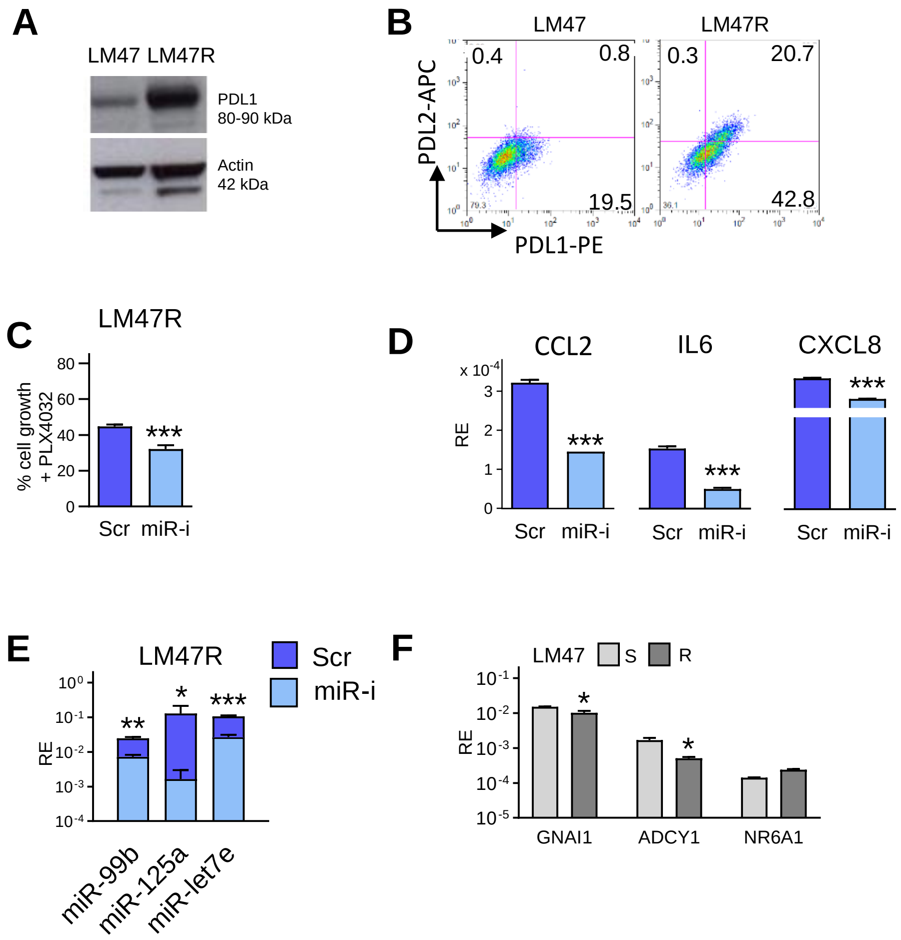

Supplementary Figure 3. miR-99b~125a~let-7e inhibition impairs growth and inflammatory signaling in BRAF/MEKi resistant melanoma cells. **(A)** PDL1 and PDL2 expression by BRAF/MEKi-sensitive (LM47) and resistant (LM47R) melanoma cell lines, evaluated by western blotting and **(B)** by flow cytometry. **(C)** Concomitant inhibition of miR-99b, miR-125a, and let-7e (miR-i) reduces cell growth compared to scrambled control (Scr), as measured by CCK8 viability assay. **(D)** Treatment with miR-inhibitors decreases expression of the transcripts of pro-inflammatory cytokines CCL2, IL6, and CXCL8. **(E)** Downregulation of miR-99b, miR-125a, and let-7e expression levels following inhibition (miR-i) compared to scrambled control (Scr). **(F)** Downregulation of GNAI1 and ADCY1 transcripts in LM47R compared to LM47 cell line, as assessed by qRT-PCR. RE: Relative Expression. *: $p<0.05$, **: $p<0.01$, ***: $p<0.001$ by Student's unpaired t test.

Supplementary Figure 3

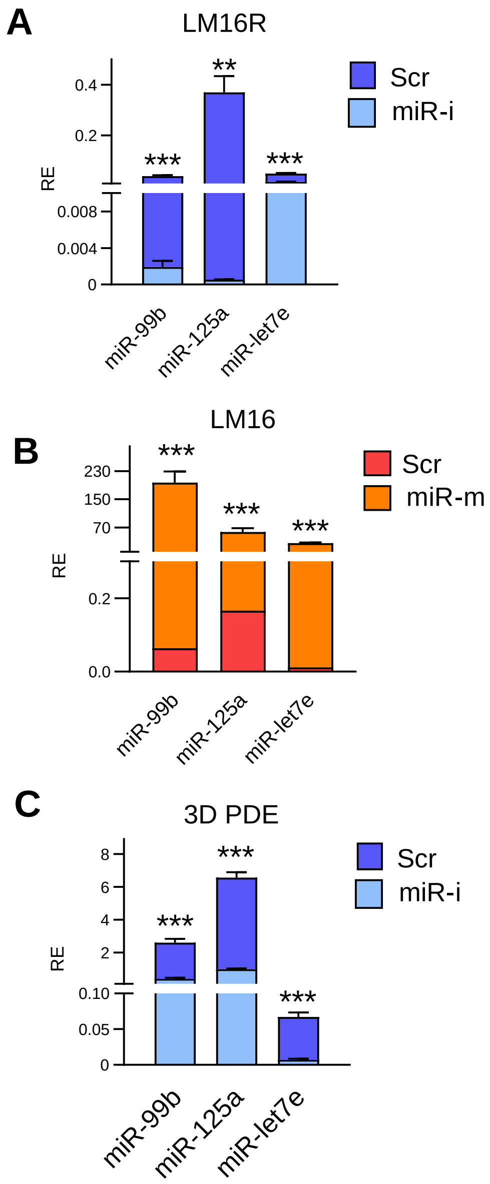

Supplementary Figure 4. qRT-PCR analysis of miR-99b, miR-125a, and let-7e expression levels under different experimental conditions. **(A)** Downregulation of miR-99b, miR-125a, and let-7e following treatment with specific miRNA inhibitors (miR-i) compared to scrambled control (Scr) in the LM16R cell line. **(B)** Upregulation of miR-99b, miR-125a, and let-7e upon treatment with specific miRNA mimics (miR-m) or scrambled control (Scr) in the LM16 cell line. **(C)** Downregulated expression levels of miR-99b, miR-125a, and let-7e in melanoma 3D PDE after 72h culture in bioreactor with specific miRNA inhibitors (miR-i) or scrambled control oligo (Scr). RE: Relative Expression. **: $p<0.01$, ***: $p<0.001$ by Student's unpaired t test.

Supplementary Figure 4
